# Controlled Cultivation Confers *Rhodiola rosea* Synergistic Activity on Muscle Cell Homeostasis, Metabolism and Antioxidant Defense in Primary Human Myoblasts

**DOI:** 10.3390/antiox13081000

**Published:** 2024-08-18

**Authors:** Fortuna Iannuzzo, Elisabetta Schiano, Arianna Pastore, Fabrizia Guerra, Gian Carlo Tenore, Ettore Novellino, Mariano Stornaiuolo

**Affiliations:** 1 Department of Pharmacy, University of Chieti-Pescara G. D’Annunzio, 66100 Chieti, Italy; fortuna.iannuzzo@unich.it; 2 Inventia Biotech-Healthcare Food Research Center s.r.l., Strada Statale Sannitica KM 20.700, 81020 Caserta, Italy; elisabettaschiano@inventiabiotech.com (E.S.); fabrizia.guerra@inventiabiotech.com (F.G.); ettorenovellino@inventiabiotech.com (E.N.); 3 Department of Pharmacy, University of Naples Federico II, Via Domenico Montesano 59, 80131 Naples, Italy; arianna.pastore@unina.it (A.P.); giancarlo.tenore@unina.it (G.C.T.)

**Keywords:** *Rhodiola rosea* L., muscle health, antioxidant activity, nutraceuticals, controlled cultivation

## Abstract

*Rhodiola rosea* L. is recognized for its adaptogenic properties and ability to promote muscle health, function and recovery from exercise. The plethora of biological effects of this plant is ascribed to the synergism existing among the molecules composing its phytocomplex. In this manuscript, we analyze the activity of a bioactive fraction extracted from *Rhodiola rosea* L. controlled cultivation. Biological assays were performed on human skeletal myoblasts and revealed that the extract is able to modulate in vitro expression of transcription factors, namely Pax7 and myoD, involved in muscle differentiation and recovery. The extract also promotes ROS scavenging, ATP production and mitochondrial respiration. Untargeted metabolomics further reveals that the mechanism underpinning the plant involves the synergistic interconnection between antioxidant enzymes and the folic/acid polyamine pathway. Finally, by examining the phytochemical profiles of the extract, we identify the specific combination of secondary plant metabolites contributing to muscle repair, recovery from stress and regeneration.

## 1. Introduction

*Rhodiola rosea* L. (*genus Rhodiola* L., Crassulaceae family) is a plant with renowned phytopharmaceutical potential (some of them documented since ancient Greek times) [1]. Twenty of the approximately one hundred Rhodiola species are commonly used as adaptogens (substances that help the human body cope with stress), mostly to enhance physiological resilience and support normal bodily functions [2,3]. In virtue of their potential to promote endurance and improve physical performance during prolonged exercise, extracts of *Rhodiola rosea* L. are widely used as dietary supplements in the sports industry [4]. The extracts also exhibit antioxidant [5], anti-fatigue [6], anti-inflammatory [7], and antidepressant [8] activities, all properties that help athletes during training and competition. Tinsley et al. have recently reviewed pre-clinical and clinical studies where, in the context of exercise performance, *Rhodiola rosea* L. extracts have been tested as an adaptogen [2]. *Rhodiola rosea* L. supplementation supports prolonged exercise by increasing mitochondrial ATP production [9]. Furthermore, chronic supplementation promotes the accumulation of hepatic glycogen at rest and concomitant attenuation of muscle glycogen depletion during exercise, implying that changes in glycogen turnover may potentially contribute to the ergogenic effects of the extracts [10]. Additionally, supplementation with *Rhodiola rosea* L. reduces post-exercise fatigue, improves tissue oxygenation and promotes the expression of proteins involved in lipid metabolism [11,12]. Finally, the antioxidant properties of *Rhodiola rosea* L. increase the expression of antioxidant enzymes, counteracting oxygen free radicals and lipid peroxidation that occurs after exercise [13].

Pharmacological studies on *Rhodiola rosea* L. are numerous. The medicinal activities of *Rhodiola rosea* L. are attributed to its phytochemicals profile, especially to phenylethanoids and phenylpropanoids present in its roots and their glycosidic derivatives [14]. Salidroside and Rosavins (a collective name for Rosavin, Rosarin, and Rosin) are among the active components of *Rhodiola rosea* L. bioactive extracts [15]. Salidroside and Rosavins contributed to the adaptogenic properties of *Rhodiola rosea* L. [14], while antioxidant activity is primarily due to organic acids and flavonoids [16]. *Rhodiola rosea* L. phytochemicals allow the plant to adapt and grow at high altitudes and in cold regions of the globe (Arctic regions of Europe, Asia, and North America). However, growing market demand has led to the development of *Rhodiola rosea* L. controlled cultivations in other regions of the globe. The phytochemical profile of controlled cultivations presents similar Salidroside and Rosavins content compared to wild harvests [17,18]. The United States Pharmacopeia standards are products titrated to 0.3% in rosavins and 0.08% in salidroside [19]. According to the Russian State Pharmacopeia, the content of rosavins should be not less than 1%, and salidroside should be not less than 0.8% [20]. The Australian standard for the extract is not less than 1.8% phenylpropanoids, 1.2% rosavins, and 0.6% salidroside [21].

The molecular mechanism underpinning *Rhodiola rosea* L. is complex, and not all of its biological activities can be attributed to salidroside or rosavins. Recently, Liu et al. (2024) demonstrated that pure salidroside might, in part, contribute to the adaptogenic effect of the extracts by effectively attenuating cardiac dysfunction, myocardial hypertrophy, myocardial fibrosis, and cardiac inflammation in animal models [22]. In other scientific settings, pure Salidroside and Rosavins failed to recapitulate *Rhodiola rosea* L. effects. This is probably to be ascribed to a synergistic effect of the phytocomplex of the plant. About 140 organic compounds have been isolated in *Rhodiola rosea* L., including polyphenols, organic acids, sugars, tannins, terpenes, and essential oils, with the root containing over 100 chemical compounds [23]. These bioactive constituents synergistically contribute to the adaptogenic properties. This synergy makes each different *Rhodiola rosea* L. cultivation of particular interest since specific climatic and cultivation conditions could generate a phytochemical profile endowed with specific properties and physiological effects [24].

In this manuscript, we analyze the biological effect of a *Rhodiola rosea* L. extract from plants harvested from controlled cultivation (RRcc) plots situated at altitudes ranging from 20 m to 600 m above sea level in Central Europe from a cultivation line called Rhodiofarm (Pharmaplant Germany a Martin Bauer company). The bioactive effects were investigated on human skeletal myoblasts and included the analysis of pathways involved in muscle differentiation and renewal, ROS scavenging, ATP production and mitochondrial respiration. The bioactive effects of RRcc were compared with those of *Rhodiola rosea* L. wild harvest (RRwh).

## 2. Materials and Methods

### 2.1. Rhodiola rosea L. Wild Harvest (RRwh) and Controlled Cultivation (RRcc)

For *Rhodiola rosea* L. wild harvest (RRwh), plants were collected in a region that straddles the borders of Kazakhstan, China and Mongolia at altitudes ranging from 2000 to 3000 m. Once collected, the roots were washed, cut into thin pieces and artificially dried to preserve medicinal properties. For *Rhodiola rosea* L. controlled cultivation (RRcc), *Rhodiola rosea* L. seeds were cultivated in specialized, isolated plots (known as Rhodiofarm, Pharmaplant Germany a Martin Bauer company (Martin Bauer, Alveslohe, Germany) to ensure pure lineage. The process was conducted under strict conditions to maintain the genetic quality and vitality of the seeds, selecting mother plants based on criteria such as disease resistance, growth rate, and active compound content. From the seeds, seedlings were raised under controlled conditions within greenhouses. This stage was crucial for ensuring a high germination rate and fostering the healthy growth of young plants. Once the seedlings had reached a suitable size, these were transplanted into cultivation plots situated at altitudes ranging from 20 m to 600 m above sea level in Central Europe. Cultivation sites were selected considering soil quality, sunlight exposure and irrigation needs to create optimal growing conditions. During the whole process, conditions such as lighting, temperature and humidity were controlled to promote the development of robust and vigorous plants. The harvest occurred three years after planting, timed to coincide with the peak in the plants’ maturity and active compound concentration. Post-harvest, the roots of Rhodiola were washed, coarsely cut and subjected to artificial drying.

### 2.2. Rhodiola rosea L. Extracts

All extracts were prepared by Finzelberg GmbH & Co. KG, Andernach/Germany, under full GMP conditions. *Rhodiola rosea* L. roots, from both controlled cultivation and wild collection, were initially prepared by cutting them to a size suitable for equipment. An exhaustive extraction was then conducted with ethanol 70% *v*/*v* using multi-stage extraction, specifically employing percolation. This method ensured thorough extraction of the bioactive compounds present in the Rhodiola root. The solvent-to-solid ratio utilized in the extraction process was 1:12. Following extraction, the eluate obtained from the process was subjected to evaporation and homogenization to yield the soft extract. This soft extract was then further processed by adding excipients (maltodextrin + silicium dioxide). These excipients were carefully chosen to enhance the stability and handling properties of the final extract. After the addition of excipients, the extract was dried by using a vacuum belt dryer and subsequently adjusted with excipients to achieve the desired composition. Lastly, the dried extract underwent additional processing steps, including grinding, mixing, sieving, and packing.

### 2.3. Cell Culture Experiments

Human skeletal muscle myoblasts (CC-2580; Lonza (Basel, Switzerland) were cultured in Dulbecco’s modified Eagle Medium (DMEM) (Gibco, (Calrsbad, CA, USA)), supplemented with 10% Fetal Bovine Serum (FBS) (Gibco, (Calrsbad, CA, USA)), 2 mM Glutamine, 100 U mL^−1^ Penicillin and 100 μg mL^−1^ Streptomycin (Gibco) and incubated at 37 °C and 5% CO_2_. These conditions were consistent throughout the experiments to ensure reproducibility and maintain the physiological relevance of the in vitro model. Cell confluence was maintained at 60–80%, and subculturing was performed at a 1:10 ratio twice a week using 25 cm^2^ flasks (Nunc, (Koln, Germany)). To induce differentiation, cells were cultivated in DMEM supplemented with 2% (*v*/*v*) Horse Serum (HS) for 7 days.

#### 2.3.1. In Vitro Treatment of Cell Cultures

Primary cultures were seeded at a density of 2 × 10^5^ cells/mL in 25 cm^2^ flasks. For cell culture experiments, the dried extract, obtained as described in Section 2.2, was solubilized using dimethyl sulfoxide (DMSO). Subsequently, the solution was diluted with water to achieve a final concentration of 30 μg/mL, maintaining a final concentration of 1% DMSO. Upon 16 h from plating, cells were treated with either RRwh or RRcc extracts. The treatments were maintained for 48 h. Untreated cells and cells treated with equal volumes of vehicles (DMSO) were used as negative controls. Upon treatment, the medium was removed, and cells were rinsed several times in phosphate-buffered saline (PBS, composed of 137 mM sodium chloride, 2.7 mM potassium chloride, 10 mM phosphate buffer, pH 7.4) to be then processed for metabolomic analysis. When indicated, cells were supplemented with Folic acid 5 μM, Cystine (Cys2) 1 mM, Quercetin (Q, 1 μM), Quercetin 3-glycoside (QG, 1 μM), Rutin (Q2G, 1 μM), and Quercetin 3-rhamnoglucoside 7-glucoside (Q3G, 1 μM), all from Merck (Darmstadt, Germany).

#### 2.3.2. Measurement of Intracellular ATP Content

Primary myoblasts were seeded into a 96-well plate at a density of 2 × 10^4^ cells/well in 100 μL of growth medium. Upon incubation with test substances for 48 h, intracellular ATP content measurement was performed using an ATP assay kit (Toyo Ink (Tokyo, Japan)), following the manufacturer’s instructions. First, cells were washed twice with PBS. Next, 50 μL of serum-free DMEM and 50 μL of ATP assay reagent were added to each well. After 15 min of incubation, cells were washed twice with PBS to remove any residual media and unmetabolized substances. Subsequently, a lysis buffer from the ATP assay kit was added to the cells. The total cellular ATP content was measured by light emission with the Envision 2105 spectrofluorometer, Perkin Elmer, Waltham, MA, USA.

#### 2.3.3. Measurement of Mitochondrial Potential

Mitochondria staining of primary myocytes was achieved by incubation with MitoTracker^®^Red CMXRos (Thermo Fisher Scientific). A dye working solution was prepared by diluting a stock solution (10 μM in DMSO) in DMEM to yield a final concentration of 100 nM. For staining of in vitro samples, cells were rinsed twice in PBS before adding the dye. Thus, cells were incubated in the presence of the probe for 40 min in a cell incubator at 37 °C and 5% CO_2_. At the end of the incubation, cells and tissues were rinsed three times in DMEM and once in PBS, then fixed in 4% formaldehyde. The quantitative measurement of MitoTracker^®^Red CMXRos fluorescence was performed with the spectrofluorometer Envision 2105 from Perkin Elmer.

#### 2.3.4. Metabolomic Profiling of In Vitro Cultured Human Myoblasts

Metabolites were extracted as follows: cell pellets were thawed on ice and 100 μL of ice-cold MeOH/H_2_O (80:20 *v*/*v*) was added. The samples were extracted in an ultrasonic bath for 6 min, vortexed for 30 s, and finally centrifuged at 14,000 rpm for 10 min at 4 °C. The supernatants were dried under nitrogen and were reconstituted in 100 μL of ACN/H_2_O (70:30) (*v*/*v*) before HRMS analysis. A pooled quality control (QC) sample was prepared by pooling an aliquot of cellular extract from each sample. Unless otherwise described, all solvents and additives were LCMS grade and purchased by Merck (Darmstadt, Germany). HRMS analyses were performed on a Thermo Ultimate RS 3000 coupled online to a Exploris 120 hybrid quadrupole Orbitrap mass spectrometer (Thermo Fisher Scientific, Bremen, Germany) equipped with a heated electrospray ionization probe (HESI II). The MS was calibrated by Thermo calmix PierceFlexmix^TM^ calibration solutions in both polarities. The analysis of cellular metabolites was performed in HILIC mode, with an Acquity BEH Amide (100 × 2.1 mm; 1.7 μm) protected with a VanGuard amide precolumn (5 × 2.1 mm; 1.7 μm) (Waters, Milford, MA, USA). The column temperature was set at 45 °C, and the flow rate was 0.400 mL/min. The mobile phase was (A) 10 mM CH_3_COONH_4_ in H_2_O/ACN (95:5 *v*/*v*) and (B) 10 mM CH_3_COONH_4_ in H_2_O/ACN (5:95 *v*/*v*). Full MS (80–800 *m*/*z*) and data-dependent MS/MS were performed at a resolution of 60,000 and 15,000 FWHM, respectively, and normalized collision energy (NCE) values of 10, 30 and 50 were used. Source parameters were as follows: sheath gas pressure, 40 arbitrary units; auxiliary gas flow, 15 arbitrary units; spray voltage, +3.5 kV, −2.8 kV; capillary temperature, 300 °C; auxiliary gas heater temperature, 280 °C. Data were analyzed with Compound Discoverer 3.3 (Thermo) and MetaboAnalyst 5.0.

#### 2.3.5. Isolation of RNA, cDNA Synthesis and qPCR Analysis Myoblast mRNAs

Total RNA was extracted according to the manufacturing protocol of RNeasy Plus Mini Kit (# 74136, Qiagen (Hilden, Germany)). First, 2 μL of total RNA was quantified by NANODROP2000 spectrophotometer (Thermo Fisher). Then, 2 μg of total RNA was converted into cDNA using SuperScript VILO Master Mix (# 11755050, Thermo Fisher) as indicated by the manufacturer, and qPCR analysis was performed using QuantiNova SYBR PCR Master Mix (# 208252, Qiagen (Hilden, Germany)). The amplification was performed using the Real-Time PCR System StepOne-Plus, Applied Biosystems (Thermo Fisher), with the following cycling conditions: 95 °C for 3 min followed by 40 cycles of 30 s of denaturation at 95 °C, 30 s of annealing at 52 °C, and 30 s product amplification at 60 °C. The relative gene expression analysis of target genes was conducted in comparison with the β-actin housekeeping control gene following the comparative 2^−ΔCt^ method. Primers used for qPCR analysis were all from Biorad (Hercules, CA, USA). Primers for myogenic differentiation 1 (myoD) (qHsaCED0023842), paired box 7 (Pax7) (qHsaCID0016103) and myogenin (qHsaCED0043933), as well as primers for gapdh; superoxide dismutase (SOD1); catalase (CAT); glutathione peroxidase (GPX1) and glutathione reductase (GR); peroxiredoxins (PRDX1); thioredoxin (TXN) and thioredoxin reductase (TXNRD1); and heme oxygenase-1 (HO-1) all belonged to the Oxidative Stress and Antioxidant Defense preassembled primer plate H96 code #10034219 from Biorad.

### 2.4. Phytochemical Profiling of Rhodiola rosea L. by UHPLC Analysis

10 mg of *Rhodiola rosea* L. extracts were solubilized in 1 mL of ethanol/H_2_O (70:30 *v*/*v*). Each sample was filtered through a 0.45 μm membrane filter and analyzed by either Reverse Phase Ultra-High Performance Liquid Chromatography associated with Photodiode Array Detection (RP-UHPLC-PDA) or High-Resolution Mass Spectrometry (RP-UHPLC-HRMS). RP-UHPLC-PDA analyses were performed on a Shimadzu Nexera UHPLC system consisting of a CBM-20A controller, two LC-30AD dual-plunger parallel-flow pumps, a DGU-20 AR5 degasser, an SPD-M20A photodiode array detector, a CTO-20A column oven and an SIL-30AC autosampler. PDA detection parameters were a sampling rate of 12 Hz and a time constant of 0.160 s, and chromatograms were extracted at 220, 280 and 330 nm. RP-UHPLC-HRMS analysis was performed on a Thermo Ultimate RS 3000 coupled online to a Q-Exactive hybrid quadrupole Orbitrap mass spectrometer (Thermo Fisher Scientific, Bremen, Germany) equipped with a heated electrospray ionization probe (HESI II). The MS was calibrated by Thermo calmix PierceTM calibration solutions in both polarities. Separation was performed with a Luna Omega C18 100 mm × 2.1 mm, 1.6 μm (L × I.D, particle size, Phenomenex^®^, Bologna, Italy) column at a flow rate of 0.4 mL/min. The mobile phases consisted of (A) 0.1% CH_3_COOH in H_2_O and (B) 0.1% CH_3_COOH in ACN. Analysis was performed in gradient as follows: 0.01 min, 2% B; 18 min, 35% B; 20.0 min, 70% B; 22.0 min, 95% B; isocratic for 2.0 min; and returning to initial conditions in 0.1 min. The column oven was set to 40 °C, and 3 μL was injected. The same chromatographic conditions (i.e., column, flow rate and mobile phases) were used for both RP-UHPLC-PDA and RP-UHPLC-HRMS analysis. MS analyses were performed in negative ionization mode (ESI-). Full MS (100–1500 *m*/*z*) and data-dependent MS/MS were performed at resolutions of 70,000 and 15,000 FWHM, respectively, and normalized collision energy (NCE) values of 15, 25 and 30 were used. Source parameters were as follows: sheath gas pressure, 50 arbitrary units; auxiliary gas flow, 13 arbitrary units; spray voltage, +3.5 kV, −2.8 kV; capillary temperature, 310 °C; and auxiliary gas heater temperature, 300 °C. Two replicates of each sample were performed. A node-based processing workflow was custom-built in Compound Discoverer^TM^ software v.3.3 (Thermo Fisher Scientific) to search and identify metabolites. Specifically, metabolite annotation was based on accurate mass measurement, MS/MS fragmentation pattern, and comparison within silico spectra with MS database searching. Salidroside was selected as an external standard for quantitative analysis. Stock solution (1 mg/mL) was prepared in 1 mL of ethanol/H_2_O (70:30). A calibration curve was obtained in the concentration range of 1–100 μg/mL (R^2^ = 0.999). For sensitivity evaluation, limits of detection (LODs) and quantification (LOQs) were calculated by the ratio between the standard deviation (SD) and analytical curve slope multiplied by 3 and 10, respectively. The compound salidroside LOD was 0.031 μg/mL and LOQ was 0.103 μg/mL.

## 3. Results

**Figure 1:**
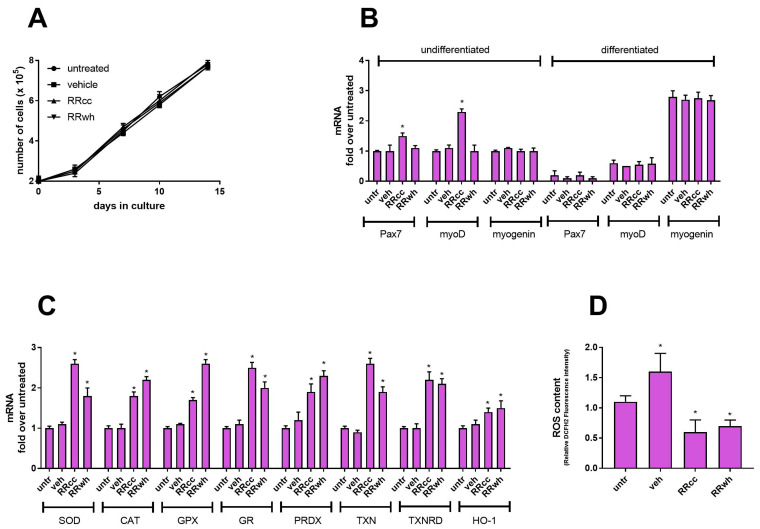
RRcc modulates undifferentiated muscle cells and promotes defense from oxidative stress. Growth (**A**), qPCR analysis of differentiation-related muscle cell transcription factors (**B**), antioxidant enzymes (**C**) and total ROS content (**D**) in primary muscle myoblasts treated for 48 h in the presence of RRcc (30 μg/mL), RRwh (30 μg/mL), an equal amount of vehicle (veh) or left untreated (untr). Data are shown as mean ± SD of five independent experiments. Statistical analysis was performed by ANOVA test comparing each mean with that of untreated cells. *p* value = * <0.05, otherwise differences were not statistically significant.

All biological experiments were conducted on primary human myoblasts supplemented with RRcc (30 μg/mL). As controls, treatment with RRwh (30 μg/mL), untreated cells (untr) and cells treated with the same amount of vehicle DMSO (veh) were analyzed in parallel. Primary human myoblasts were very sensitive to DMSO. However, treatments of up to 30 μg/mL of RRcc and RRwh did not change the overall growth rate of the cell cultures, with myoblasts presenting an equal degree of confluency at the end of every experiment (Figure 1A).

### 3.1. RRcc Promotes the Transcription of myoD and Pax7

First, we analyzed the effect of RRcc on the expression of differentiation markers of skeletal muscle cells. Pure salidroside has been shown to maintain murine skeletal muscle cells in an undifferentiated state by promoting the expression of transcription factors involved in muscle recovery and regeneration [25]. The quantitative measurement of salidroside by UHPLC-LRMS revealed that RRcc and RRwh contain 5.724 ± 0.170 μg/mg (~0.8% *w*/*w*) and 9.136 ± 0.192 μg/mg (~1.4% *w*/*w*) salidroside, respectively. In terms of the effect on the differentiation of skeletal muscle cells, the two extracts should have had similar effects. However, analyzed by qPCR, treatment with RRcc but not with RRwh promoted the upregulation of two mRNA markers of muscle cell undifferentiation: myogenic differentiation 1 *myoD* (a marker of proliferating undifferentiated myoblasts) and paired box 7 *Pax7* (a marker of undifferentiated reserve muscle satellite cells) (Figure 1B). In vivo, Pax7-positive cells are placed underneath the myofiber basal lamina, where they act as a muscle stem cell reservoir and allow the repair and maintenance of myofibers. When cultivated for 6 days in the appropriate differentiation medium, primary human myoblasts spontaneously differentiate into myotubes, as shown by the appearance of the myotube marker myogenin (Figure 1B). During differentiation, supplementation with RRcc or RRwh did not alter the expression of myogenin, suggesting that the two extracts do not retard the differentiation of myoblasts into myotubes. Similarly, when cultivated in the absence of differentiation medium, RRcc and RRwh do not promote the expression of myogenin, suggesting no effect on the subpopulation of myogenin-positive cells. RRwh is thus inactive in intracellular pathways regulating muscle homeostasis. On the contrary, RRcc is active on a subpopulation of Pax7- and myoD-positive myoblast cells involved in muscle renewal. Since both Pax7 and myoD are upregulated by RRcc, and myoD is expressed at later time points during myoblast differentiation, RRcc very likely allows the bypass of early differentiation control points, promoting the proliferation of Pax7/myoD-positive myoblasts.

### 3.2. RRcc and RRwh Regulate the Redox State of Skeletal Muscle Cells

Considering that the differentiation state of muscle cells depends on their redox state [26], we wondered if the effect of RRcc on *myoD* and *Pax7* expression could be the result of a different antioxidant activity of RRcc compared to RRwh. Despite the different effect on the transcription of undifferentiation-related genes, both extracts promoted the transcription of mRNAs coding for antioxidant enzymes. As shown in Figure 1C, mRNA for superoxide dismutase (SOD), catalase (CAT), glutathione peroxidase (GPX) and glutathione reductase (GR), peroxiredoxins (PRDX), thioredoxin (TXN), thioredoxin reductase (TXNRD) and heme oxygenase-1 (HO-1) were all upregulated by both RRcc and RRwh. As a consequence, myoblasts treated with RRcc and RRwh showed decreased ROS content when compared to cells treated with vehicle, confirming that both RRcc and RRwh protect muscle cells from endogenous or culture-derived ROS.

### 3.3. RRcc and RRwh Both Promote ATP Production and Mitochondrial Activity

We thus wondered if the different effects of the two extracts might have been the result of a different metabolic consequence of their treatment.

**Figure 2:**
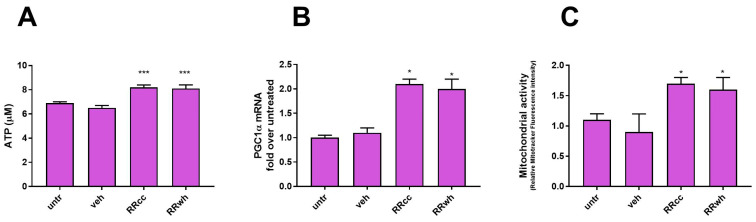
RRcc promotes ATP production and mitochondrial activity in cultured human myoblasts. Intracellular ATP content (**A**), *Pgc1α* mRNA levels (**B**) and mitochondrial activity(**C**) in primary muscle myoblasts treated for 48 h in the presence of RRcc (30 μg/mL), RRwh (30 μg/mL), an equal amount of vehicle (veh) or left untreated (untr). Data are shown as mean ± SD of five independent experiments. Statistical analysis was performed by ANOVA test comparing each mean with that of untreated cells. *p* value = * <0.05, *** < 0.001, otherwise differences were not statistically significant.

Along with the support of muscle regeneration, RR is known to promote mitochondrial activity and ATP production in muscle cells. We thus measured intracellular ATP concentration in human muscle cells treated with RRcc or RRwh by luminescence assay. As shown in Figure 2A and compared to untreated or vehicle-treated cells, 48 h of incubation with RRcc or RRwh promoted a statistically significant increase in ATP production (untr: 6.9 ± 0.1 μM; veh: 6.5 ± 0.2 μM; RRcc: 8.2 ± 0.2 μM; RRwh: 8.1 ± 0.3 μM; *** = ANOVA *p* value < 0.001; *n* = 3 independent experiments). However, no statistically significant difference was measured between RRcc and RRwh in terms of ATP production.

**Figure 3:**
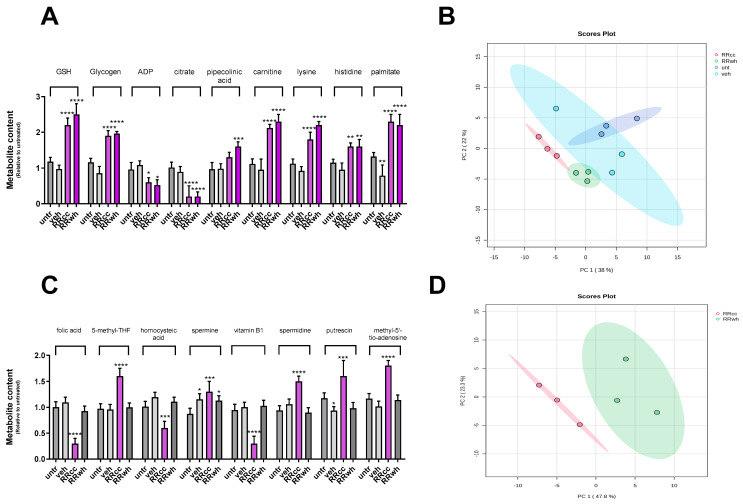
RRcc presents folic acid-cycle and polyamine pathway modulatory activity in human myoblasts. Metabolic profile of primary muscle myoblasts treated for 48 h with RRcc (30 μg/mL), RRwh (30 μg/mL), an equal amount of vehicle (veh) or left untreated (untr). (**A**) Content (fold variation vs. untr) of the indicated metabolites as measured in the four different experimental conditions; (**B**) primary component analysis (PCA) score plot showing that the metabolic profiles of RRcc and RRwh are different from veh and untr cells; (**C**) content (fold variation vs. untr) of the folic acid-cycle and polyamine pathway metabolites as measured in the four different experimental conditions (*n* = 3 independent experiments); (**D**) PCA score plot showing the metabolic profile of RRcc being different from RRwh. In A and C, values are represented as mean ± SD. Two-way ANOVA and Bonferroni post-test analysis were performed; * = *p* < 0.05; ** = *p* < 0.01; *** = *p* < 0.001; **** = *p* < 0.0001; otherwise differences were not statistically significant.

In order to confirm the mitochondrial boosting potential of the two extracts, the transcriptional upregulation of *Pgc1α* (a master regulator of mitochondrial activity) and the intermembrane mitochondrial potential were measured in RRcc- and RRwh-treated cells. Both the extracts strongly upregulated *Pgc1α.* The intermembrane mitochondrial potential was measured with the mitochondrial probe Mito Tracker CMX-ROS. The dye accumulates in mitochondria depending on their membrane potential and, thus, on their activity. As shown in Figure 3B, when analyzed by fluorimetry, human myoblast cells treated with RRwh or RRcc showed increased mitochondrial activity compared to vehicle-treated cells (untr: 1.1 ± 0.1-fold increase in fluorescence; veh: 0.9 ± 0.3; RRcc: 1.7 ± 0.1; RRwh: 1.6 ± 0.2; * = ANOVA *p* value < 0.05; *n*= 3 independent experiments), confirming that both RRcc and RRwh stimulate mitochondrial activity. No difference between RRcc and RRwh in terms of PGC1α mRNA levels or intermembrane mitochondrial potential could be detected.

So far, differently from RRwh, RRcc presents an unprecedented regulatory activity on transcription factors involved in the maintenance of undifferentiated myoblasts Pax7 and myoD. However, this peculiar effect of RRcc does not depend on a different antioxidant, ATP-stimulatory, mitochondriogenic activity of the extract.

### 3.4. Metabolomic Profiling of RRcc- and RRwh-Treated Human Myoblasts

To investigate the metabolic pathways selectively modulated by RRcc in muscle cells, we performed HR mass-spectrometry metabolic profiling of cells undergoing treatment for 48 h with 30 μg/mL of RRcc. For comparison, metabolic profiling was also performed in RRwh (30 μg/mL), vehicle and untreated cells as controls. One hundred and ninety metabolites were unambiguously identified by comparison with spectral data from an in-house library of standards from HMDB. As shown in Figure 3, principal component analysis (PCA) showed that RRcc- and RRwh-supplemented cells were clearly clustered from untr and veh cells. The metabolite fluctuation (Figure 3A) indicates specific metabolites (reduced GSH, glycogen, ADP, citrate, DL-Pipecolinic acid, L-Carnitine, L-Lysine, L-Histidine and palmitic acid) that are strongly associated with the separation of both RRcc and RRwt from untreated cells. The increase in reduced GSH (untr: 1.18 ± 0.12; veh: 0.97 ± 0.11; RRcc: 2.2 ± 0.2; RRwh: 2.5 ± 0.3; fold difference compared to untr) strongly confirms the antioxidant potential of both RRcc and RRwh extracts. The decrease in intracellular glycogen (untr: 1.16 ± 0.11-fold increase in fluorescence; veh: 0.86 ± 0.19; RRcc: 1.90 ± 0.15; RRwh: 1.96 ± 0.06) proves that RRcc and RRwh promote glycogen production in muscle cells, at least in vitro. The decrease in ADP (untr: 0.95 ± 0.20-fold increase; veh: 1.08 ± 0.12; RRcc: 0.60 ± 0.13; RRwh: 0.52 ± 0.15) induced by the RR extracts confirms the increase in ATP measured by luminescence, confirming the energy-stimulating activity of the extract in muscle cells.

Moreover, RRcc and RRwh treatment both cause an increase in intracellular levels of both L-Lysin (untr: 1.12 ± 0.13; veh: 0.92 ± 0.12; RRcc: 1.80 ± 0.20; RRwh: 2.2 ± 0.1) and L-Histidine (untr: 1.14 ± 0.10; veh: 0.95 ± 0.19; RRcc: 1.60 ± 0.10; RRwh: 1.60 ± 0.20) that reveals the sparing of intracellular substrates for energy production. Lysin sparing may also be supported by increased levels of its non-enzymatic by-product DL-Pipecolinic acid (untr: 0.97 ± 0.19; veh: 0.98 ± 0.14; RRcc: 1.30 ± 0.14; RRwh: 1.60 ± 0.13). Substrates of acylcarnitines production—L-Carnitine (untr: 1.12 ± 0.14; veh: 0.95 ± 0.30; RRcc: 2.12 ± 0.10; RRwh: 2.30 ± 0.2) and palmitic acid (untr: 1.32 ± 0.11; veh: 0.78 ± 0.30; RRcc: 2.3 ± 0.2; RRwh: 2.2 ± 0.3)—both increase, suggesting the sparing of fatty acids for mitochondrial metabolism. Finally, citrate (untr: 1.02 ± 0.13; veh: 0.89 ± 0.15; RRcc: 0.20 ± 0.3; RRwh: 0.20 ± 0.13) decreases in both RRcc and RRwh cells, suggesting the promotion of the Krebs cycle and, most likely, the inhibition of the mevalonate pathway.

### 3.5. RRcc Presents Folic Acid Cycle Modulatory Activity

As shown in Figure 3D, PCA demonstrated that RRcc-supplemented cells grouped distinctly from RRwh cells. Metabolite fluctuation (Figure 3C) revealed that specific metabolites (folic acid, 5-methyl-THF, homocysteic acid, spermidine, vitamin B1, putrescein, methyl-5′-tio-adenosine) were strongly associated with the separation of RRcc from RRwt. In RRcc cells, folic acid (untr: 1.02 ± 0.10; veh: 1.09 ± 0.10; RRcc: 0.30 ± 0.10; RRwh: 0.92 ± 0.09), 5-methyl-THF (untr: 0.96 ± 0.09; veh: 0.95 ± 0.09; RRcc: 1.60 ± 0.15; RRwh: 0.99 ± 0.2) and vitamin B1 (untr: 0.94 ± 0.11-fold increase; veh: 1.00 ± 0.09; RRcc: 0.30 ± 0.14; RRwh: 1.02 ± 0.11) are decreased, suggesting that RRcc stimulates the folate pathway. Specifically, the significant reduction in vitamin B1 observed in RRcc compared to RRwh could be attributed to the stimulatory effect of RRcc on the folate-mediated one-carbon metabolism, which is linked to the pentose phosphate pathway, where thiamine serves as a critical cofactor. This seems to be further confirmed by the relative levels of homocisteic acid (untr: 1.00 ± 0.09-fold increase; veh: 1.19 ± 0.09; RRcc: 0.60 ± 0.13; RRwh: 1.10 ± 0.08). Similarly, RRcc (but not RRwh) stimulates four metabolites of the polyamine pathway: spermine (untr: 0.87 ± 0.10; veh: 1.15 ± 0.10; RRcc: 1.30 ± 0.20; RRwh: 1.12 ± 0.09), spermidine (untr: 0.93 ± 0.09-fold; veh: 1.05 ± 0.10; RRcc: 1.50 ± 0.10; RRwh: 0.89 ± 0.09), putrescine (untr: 1.17 ± 0.10; veh: 0.93 ± 0.07; RRcc: 1.60 ± 0.30; RRwh: 0.98 ± 0.11) and methyl-5-tioadenosine (untr: 1.16 ± 0.09; veh: 1.01 ± 0.10; RRcc: 1.80 ± 0.10; RRwh: 1.13 ± 0.09).

Together with metabolically promoting redox maintenance, ATP and protein production, RRcc presents an unprecedented stimulatory activity on folate-mediated one-carbon metabolism, absent in RRwh and resulting in an intracellular increase in polyamines.

### 3.6. Both Folic Acid Stimulatory and Antioxidant Activity Contribute to RRcc Activity

**Figure 4:**
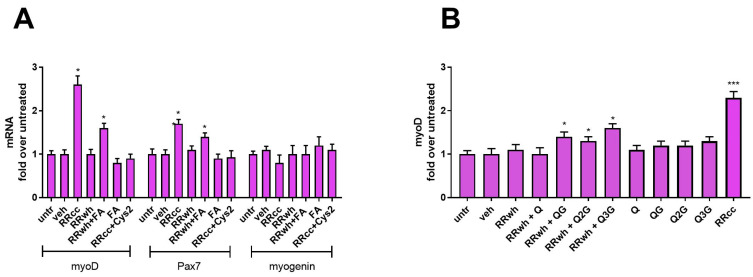
RRcc activity relies on the synergism between the folic acid pathway and antioxidant activity and on the presence of quercetin-glycosides. (**A**,**B**) mRNA levels of muscle cell differentiation markers in primary muscle myoblasts treated for 48 h in the presence of RRcc (30 μg/mL), RRwh (30 μg/mL), an equal amount of vehicle (veh) or left untreated (untr). When indicated, folic acid (FA, 5μM), cystine (Cys2, 1 mM), quercetin (Q, 1 μM), quercetin 3-glycoside (QG, 1 μM), rutin (Q2G, 1 μM) and quercetin 3-rhamnoglucoside 7-glucoside (Q3G, 1 μM) were included in the treatment. Data are shown as mean ± SD of five independent experiments. Statistical analysis was performed by ANOVA test comparing each mean with that of untreated cells. *p* value = * <0.05, *** <0.001, otherwise differences not statistically significant.

In order to verify whether folic acid pathway stimulatory activity was indeed the key element responsible for the difference between RRcc and RRwh, we treated skeletal muscle cells with RRwh in the presence of folic acid. As shown in Figure 4, the presence of 5 μM folic acid indeed confers on RRwh the ability to upregulate the mRNA of myoD and Pax7 in skeletal muscle cells. Surprisingly, 5 μM folic acid alone failed in promoting the transcription of the two genes, suggesting that the other pathways stimulated by RRcc synergize for the overall activity of the extract. Since antioxidant activity was the other pathway mainly stimulated by the RRcc, we attempted inhibition of the latter by co-supplementation with pro-oxidant molecule cystine (Cys2), given at the concentration of 1 mM. Indeed, both RRcc and RRwh supplemented with folic acid lost their activity in upregulating myoD and Pax7 in the presence of the pro-oxidant cystine, confirming that RRcc relies on the synergistic effect of folic acid and antioxidant pathways to exert its effect on the differentiation state of muscle cells (Figure 4).

### 3.7. Phytochemical Profile of RRcc vs. RRwh

**Figure 5:**
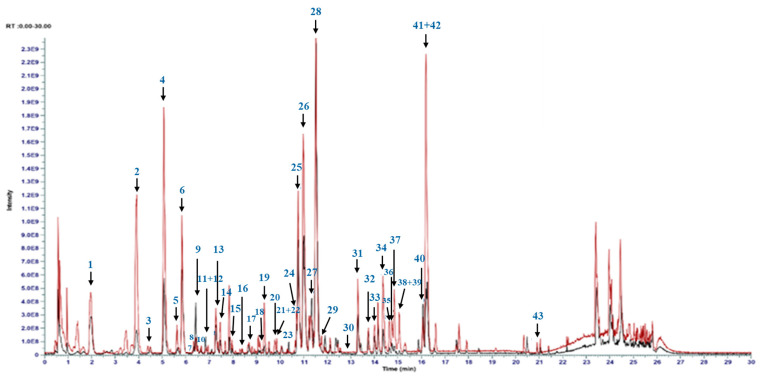
Overlapped UHPL-HRMS base peak chromatogram of *Rhodiola rosea* L. wild harvest (RRwh, black chromatogram) and *Rhodiola rosea* L. controlled cultivation (RRcc, red chromatogram) with peak annotation.

In order to identify the phytochemical profile responsible for the RRcc activity, we analyzed its bioactive fraction by high-resolution mass spectrometry. Untargeted metabolomics (Figure 5) allowed the identification of a further 42 metabolites (Table 1). Compared to RRwh, RRcc is enriched in various chemical classes of metabolites (Table 2), including the non-cyanogenic hydroxynitriles (e.g., rhodiocyanoside A (**2**)) and the cyanogenic glycosides (e.g., heterodendrin (**4**)). RRcc also demonstrates to be enriched in various classes of polyphenols, including flavanols (e.g., gallocatechin (**8**)), hydroxycinnamic acids (caffeic acid (**13**) and its hexoside (**10**), as well as ferulic acid (**23**)), flavonol (rhodiolin (**43**), and flavonol glycosides (e.g., quercetin hexoside (**22**), quercetin 3-(2G-glucosylrutinoside (**20**) and rutin (**21**)). Additional glycosides in RRcc include sacranoside A (**35**) and its isomer (**37**), along with rhodiolatuntoside (**36**). The flavanone eriodictyol (**38**), the flavone C-glycoside vicenin 2 (**39**) and rhodiooctanoside (**41**), belonging to the class of acyclic alcohol glycosides, are also found in higher amounts compared to RRwh (Figure 5). On the contrary, RRcc contains—in lower quantities—cyanogenic glycoside and rosiridin (**28**), as well as glycosides such as rosin (**27**), rhodioloside C (**40**), rhodioloside D (**9**), salidroside hexoside (**7**) and rhodioloside A (**12**). The hydroxycinnamic acid, coumaric acid (**5**), and the flavanol catechin (**11**) are present in reduced quantities as well. Additionally, a rosiridin isomer (**29**) and the gallic acid derivative sachalinoside A (**33**) are also lower in RRcc.

Considering the enrichment of quercetin glycosides found in RRcc compared to RRwh, we wondered if these glycosylated quercetins could be contributing to the RRcc effect on the myoD-positive population of myoblasts. As shown in Figure 4B, RRwh supplemented with 1 μM quercetin glycosides (quercetin 3-glucosides, rutin, quercetin 3-O-rutinoside-7-O-glucoside) but not with 1 μM quercetin aglycone presented myoD-promoting activity in myoblasts. Interestingly, when supplemented alone, the three quercetin glycosides presented a weaker potency compared to RRcc, indicating that they cannot fully recapitulate the extract and pointing instead toward a synergistic effect among different compounds in the RR extract being responsible for it.

## 4. Discussion

*Rhodiola rosea* L., recognized for its adaptogenic properties, has shown promising results in muscle health due to its influence on key biological pathways that are crucial for muscle function and recovery [3]. Scientific evidence indicated that *Rhodiola rosea* L. may enhance physical endurance and aid in muscle repair, possibly due to its effects on stress-response systems and increase in antioxidant capacity [4]. Moreover, its bioactive components are thought to mitigate fatigue and improve recovery times, making it of interest to athletes and those undergoing physical rehabilitation [27]. Interestingly, the evaluation of specific results obtained from the two plant matrices herein tested, i.e., RRcc and RRwh, adds depth to the discussion on novel mechanisms through which *Rhodiola rosea* L. may exert its beneficial effects on muscle health. In this regard, the results showed that RRcc led to the activation of folate-mediated one-carbon metabolism and polyamine biosynthesis, especially stimulating metabolites such as spermine, spermidine and putrescine. The effect of RRcc on the polyamine pathway might also be related to the increased consumption of folic acid and vitamin B1. As shown by others [28] and by us in our manuscript, RRcc extracts transcriptionally regulate *Pax7* and *MyoD*, contributing to the maintenance of an undifferentiated pool of *MyoD*-positive myoblasts, which are important for muscle regeneration. Notwithstanding, RRcc does not impair myoblast differentiation into myotubes. The involvement of the cellular antioxidant machinery and the polyamine pathway has already been demonstrated in muscle cells [29,30]. Moreover, the polyamine pathway has been involved in many other aspects of muscle biology, including protein turnover and metabolic adaptation to anoxia [31]. In this regard, the modulation of the transcription and expression of specific genes involved in these processes, potentially induced by RRcc, warrants further investigation.

Our results also indicate that controlled cultivation conditions for RRcc may result in an improved bioactive compound profile, leading to more effective stimulation of these critical metabolic pathways. Our data point toward quercetin-glycosides as contributing to the effect of RRcc on muscle cells, at least in vitro. However, the effect of a phytocomplex is rarely recapitulated by one or few of its components and, instead, is most of the time attributable to the synergistic activity of many of its secondary metabolites. We could thus not exclude that other molecules and their synergism might contribute to RRcc’s overall activity.

Our results regarding the metabolic profile of RRcc, together with the analysis of its mechanism of action, are particularly important in the context of the modern economy and globalization. As a consequence of growing market demand, controlled cultivations of *Rhodiola rosea* L. are starting to appear in several regions of the globe. The different growing conditions (altitude, temperature, water availability) alter the phytochemical profiles of the plants and their bioactive fractions. Notwithstanding, the phytocomponents of these *Rhodiola rosea* L. controlled cultivations are rarely analyzed in detail. In most cases, this limitation results from the use of chromatographic techniques such as HPLC-DAD and from not using high-resolution instrumentation [32,33]. The study conducted by Vouillamoz et al. in 2012 that focused on the first synthetic variety of *Rhodiola rosea* L. from the Swiss Alps, called “Mattmark”, showed limitations in metabolite analysis with the quantification of only six major compounds using HPLC-DAD analysis [34]. It is noteworthy that only one previous study by Alperth et al. combined UHPLC-DAD analysis with mass spectrometry, allowing the identification of only 18 metabolites [35]. In our research, we performed a UHPLC-HRMS analysis of *Rhodiola rosea* L. RRcc and compared it to RRwh, with the unequivocal identification of 43 metabolites, including phenylpropanoids, phenylethanoids, non-cyanogenic hydroxynitriles, glycosides and polyphenols (flavanols, hydroxycinnamic acids, flavonols and flavonol glycosides). This analysis stands out from previous studies in the literature, which have mainly focused on the identification and quantification of a few key bioactive compounds, particularly salidroside and total rosavins (rosavin, rosin, rosarin) [17,36,37,38]. In this context, our study represents an innovative and significant contribution, as it is the first to provide a comprehensive analysis of the compounds present in *Rhodiola rosea* L. by combining conventional methods such as HPLC-DAD with high-resolution mass spectrometry analysis, thus providing a more detailed understanding of the metabolic profile of this medicinal plant.

Our study demonstrates that the transition from wild to controlled cultivation does not compromise the bioactive potential and the antioxidant profile of *Rhodiola rosea* extract. Moreover, while the quantities of rosavin and salidroside in RRcc remained within the limits specified by The United States Pharmacopeia [19], the controlled cultivation allowed an increase in other metabolites. This effect could be related to factors such as consistent environmental conditions, nutrient availability, and selective breeding practices in controlled cultivation settings that could potentially enhance the production of secondary metabolites with bioactive properties [17]. In this regard, the unique phytochemical profile of RRcc, enriched in various metabolites such as flavonoids, phenolic acids, and specifically polyphenolic compounds, strongly suggests its improved biological activities compared to wild harvests. These compounds are renowned for their antioxidant, anti-inflammatory, and anti-fatigue properties, which are beneficial in managing the oxidative stress and inflammatory responses implicated in the pathophysiology of sarcopenia [39]. Furthermore, based on Table 2, a comparative dose of the extracts of *Rhodiola rosea* L. can be calculated. Specifically, based on the calculated comparative doses of various compounds (e.g., rhodiocyanoside A, heterodendrin, gallocatechin and others) between RRcc and RRwh, it could be speculated that to induce an effect on folic acid and polyamine pathways, approximately a 2.5-fold higher dose of RRwh extract would be required compared to RRcc extract.

Although our study focused on metabolomic analysis and in vitro observations, which are inherently limited in confirming direct in vivo effects, this approach was crucial for identifying potential metabolic pathways involved and preliminary mechanisms of action of RRcc. Nonetheless, previous studies provided solid evidence of the beneficial effects of *Rhodiola rosea* supplementation in vivo in terms of response to muscle damage, increased antioxidant capacity and enhanced physical performance [4,40]. Therefore, the findings of this study perfectly align with this evidence, supporting the hypothesis that the metabolic changes observed in vitro could reflect similar biological effects in vivo. Moreover, compared to existing research, the obtained results not only confirm previous hypotheses about the beneficial role of *Rhodiola rosea* L. but also unveil new research directions. Based on the observed stimulatory effects of RRcc on folate and polyamine metabolic pathways, which are essential for the maintenance, growth and repair of muscle cells, it is indeed conceivable to speculate on its potential effect in the treatment of sarcopenia. Sarcopenia, characterized by progressive and generalized loss of skeletal muscle mass and strength, poses significant challenges, particularly in the aging population [41]. In this regard, the present study demonstrated the efficacy of RRcc in stimulating ATP production and improving mitochondrial respiration. This is particularly relevant for the management of sarcopenia, as improved mitochondrial function is associated with increased energy availability and efficiency in muscle cells, potentially counteracting the energy deficit reported in sarcopenic muscle [42].

## 5. Limitations of the Study

While this study offers valuable insights into the potential benefits of controlled cultivation of *Rhodiola rosea* L., various limitations must be acknowledged. First, the experimental design primarily focused on in vitro assessments of bioactivity, which may not fully capture the complex interplay of phytochemicals and physiological responses in vivo. However, muscle biology is subjected to great in vivo variability (e.g., influence of food, feeding scheme, training), and preliminary in vitro experiments serve as valuable initial steps as they help with designing the proper experimental platforms. Additionally, the scope of this study did not encompass comprehensive clinical trials to evaluate the efficacy and safety of *Rhodiola rosea* L. extracts in human subjects. Moreover, while the study identified salidroside, rosavins and quercetin glycosides as promising bioactive compounds in *Rhodiola rosea* L., their specific mechanisms of action and potential synergistic effects remain to be fully elucidated. Future research endeavors should aim to address these limitations by conducting robust clinical trials, employing advanced analytical techniques to elucidate molecular mechanisms, and optimizing cultivation protocols to ensure the consistency and reproducibility of results. Despite these limitations, the findings of this study provide a solid foundation for the further exploration of the controlled cultivation of *Rhodiola rosea* L. as a potential therapeutic agent for various health applications.

## 6. Conclusions

This study has demonstrated that controlled cultivation of *Rhodiola rosea* L. significantly enhances its bioactive potential. Our comprehensive phytochemical analysis revealed different bioactive compounds in extracts from RRcc compared to RRwh. Moreover, the properties of the RRcc extract measured on primary human myocytes revealed that, along with the expected ATP stimulatory activity, the extract derived from this specific controlled cultivation shows an unprecedented transcriptional regulation of two markers of muscle regeneration, *Pax7* and *myoD,* and a stimulatory effect on the folate and polyamine pathway, metabolic processes that play a significant role in resilience muscle regeneration during injuries or, more physiologically, as a consequence of physical exercise. Overall, the implications of our findings extend beyond the field of sports nutrition and physical rehabilitation, opening new avenues for research into the role of natural supplements in combating age-related muscle loss. Future studies could focus on clinical trials to validate these effects in human subjects and explore the molecular mechanisms underlying the observed bioactivities, potentially guiding the optimization of cultivation practices to yield even more potent health benefits.

## Figures and Tables

**Table 1:** UHPLC-HRMS analysis of phytochemical compounds identified in RRcc and RRwh extracts. Retention time, identity, *m*/*z* of molecular ions, fragments, and mass accuracy are indicated.

Peak	Rt (min)	Compound	Formula	*m*/*z* [M + H]^−^ or [M + CH_3_COO]^−^	MS/MS	Mass Accuracy (Error ppm)
**1**	1.97	Gallic acid	C_7_H_5_O_5_	169.0132	125.0232	0.47
**2**	3.91	Rhodiocyanoside A	C_11_H_16_NO_6_	318.1195 [M + CH_3_COO]^−^	258.9975; 161.0445; 59.0127	1.97
**3**	4.67	*p*-hydroxyphenacyl-β-D-glucopyranoside	C_14_H_17_O_8_	313.0922	151.039	3.71
**4**	5.2	Heterodendrin	C_11_H_18_NO_6_	320.1349 [M + CH_3_COO]^−^	261.1170; 161.0445; 59.0127	−0.07
**5**	5.6	Coumaric acid	C_9_H_7_O_3_	163.0391	119.049	0.66
**6**	5.84	Salidroside	C_14_H_19_O_7_	299.1136	179.0552; 161.0446; 119.0339	3.64
**7**	6.06	Salidroside hexoside	C_20_H_29_O_12_	461.1667	299.1137; 179.0552	2.96
**8**	6.36	Gallocatechin	C_14_H_13_O_7_	305.0667	125.0232	3.51
**9**	6.42	Rhodioloside D	C_16_H_29_O_8_	349.1855	179.0552; 119.0338	−0.51
**10**	6.66	Caffeic acid hexoside	C_15_H_17_O_9_	341.0888	179.0341	2.66
**11**	6.81	Catechin	C_15_H_13_O_6_	289.0722	169.0134	4.8
**12**	6.83	Rhodioloside A	C_16_H_27_O_8_	347.1713	179.0552; 119.0338	−4.04
**13**	7.27	Caffeic acid	C_9_H_7_O_4_	179.0342	135.044	1.39
**14**	7.47	Viridoside	C_15_H_21_O_7_	313.1249	151.0754	3.98
**15**	7.96	Unknown	-	755.2054	609.1464; 300.0274	−4.19
**16**	8.37	Epicatechin	C_15_H_13_O_6_	289.0722	169.0134	4.8
**17**	8.68	Epigallocatechine 3-gallate	C_22_H_17_O_11_	457.0781	169.0133	2.81
**18**	9.02	Quercetin hexoside isomer 1	C_21_H_19_O_12_	463.0889	300.0276	1.4
**19**	9.35	Epigallocatechin 3-gallate	C_22_H_17_O_11_	457.0781	169.0133	2.81
**20**	9.79	Quercetin 3-(2G-glucosylrutinoside)	C_33_H_39_O_21_	771.2002	463.0879; 301.0344	1.48
**21**	9.87	Rutin	C_27_H_29_O_16_	609.1461	463.0884; 300.0276	0.88
**22**	9.9	Quercetin hexoside isomer 2	C_21_H_19_O_12_	463.0887	300.0276	1.4
**23**	10.37	Ferulic acid	C_10_H_9_O_4_	193.05	178.0263	−3.16
**24**	10.7	Rhodioloside E	C_21_H_37_O_11_	465.2344	191.0554; 149.0445	−0.67
**25**	10.77	Rosavin	C_20_H_27_O_10_	487.1823 [M + CH_3_COO]^−^	427.1610; 293.0882	2.59
**26**	11.01	Rosarin	C_20_H_27_O_10_	487.1823 [M + CH_3_COO]^−^	427.1610; 293.0882	2.59
**27**	11.34	Rosin	C_15_H_19_O_6_	355.1398 [M + CH_3_COO]^−^	161.0446	−0.01
**28**	11.51	Rosiridin isomer 1	C_16_H_27_O_7_	391.1975 [M + CH_3_COO]^−^	331.1752; 179.0555; 161.0447	3.2
**29**	11.9	Rosiridin isomer 2	C_16_H_27_O_7_	391.1975 [M + CH_3_COO]^−^	331.1752; 179.0555; 161.0447	3.2
**30**	13.06	Rhodiolgin	C_21_H_19_O_12_	463.0868	317.0302	−0.67
**31**	13.31	Unknown	-	737.5194	677.4975	1.69
**32**	13.75	Sachaloside II	C_21_H_33_O_10_	505.2295 [M + CH_3_COO]^−^	445.2080;	3.17
**33**	14	Sachalinoside A	C_23_H_31_O_11_	483.1875	271.0461	1.97
**34**	14.51	Rhodiosin	C_27_H_29_O_16_	609.1466	301.0354	2.62
**35**	14.64	Sacranoside A isomer 1	C_21_H_33_O_10_	505.2295 [M + CH_3_COO]^−^	445.2080;	3.17
**36**	14.75	Rhodiolatuntoside	C_21_H_19_O_11_	447.0927	301.0352	1.017
**37**	14.86	Sacranoside A isomer 2	C_21_H_33_O_10_	505.2295 [M + CH_3_COO]^−^	445.2080;	3.17
**38**	15.1	Eriodictyol	C_15_H_12_O_6_	287.056	151.0026; 135.0404	0.97
**39**	15.18	Vicenin 2	C_27_H_29_O_15_	593.1519	285.0404	1.3
**40**	16.06	Rhodioloside C	C_22_H_37_O_12_	493.2296	447.2239	4.61
**41**	16.23	Rhodiooctanoside	C_19_H_35_O_10_	423.2239	291.1815	3.46
**42**	16.24	Rhodioloside B	C_22_H_37_O_12_	493.2296	447.2239	4.61
**43**	20.9	Rhodiolin	C_25_H_19_O_10_	479.0989	299.0199	−4.3

**Table 2:** Phytochemicals enriched in RRcc compared to RRwh.

Peak	Compound	% AUC Ration (RRcc/RRwh)
**2**	Rhodiocyanoside A	341%
**4**	Heterodendrin	185%
**8**	Gallocatechin	156%
**10**	Caffeic acid hexoside	117%
**13**	Caffeic acid	165%
**18**	Quercetin hexoside	208%
**20**	Quercetin 3- (2G-glucosylrutinoside)	362%
**21**	Rutin	186%
**22**	Quercetin hexoside	208%
**23**	Ferulic acid	227%
**34**	Rhodiosin	205%
**35**	Sacranoside A	239%
**36**	Rhodiolatuntoside	165%
**37**	Sacranoside A isomer	395%
**38**	Eriodictyol	168%
**39**	Vicenin 2	282%
**41**	Rhodiooctanoside	314%
**43**	Rhodiolin	475%

%AUC ratio (RRcc/RRwh) represents the percentage ratio of the Area Under the Curve for compounds measured in *Rhodiola rosea* L. controlled cultivation compared to wild harvest. It quantifies the relative abundance of each compound in the controlled cultivation extracts versus those in the wild, thereby indicating variances in phytochemical profiles due to cultivation conditions.

## Data Availability

The data used to support the findings of this study are included within the article.
